# Outcomes of completion lobectomy long after segmentectomy

**DOI:** 10.1186/s13019-019-0941-8

**Published:** 2019-06-26

**Authors:** Yuki Takahashi, Masahiro Miyajima, Makoto Tada, Ryunosuke Maki, Taijiro Mishina, Atsushi Watanabe

**Affiliations:** Department of Thoracic Surgery, Sapporo Medical University, School of Medicine and Hospital, South 1, West 16, Chuo-ku, Sapporo, Hokkaido Japan

**Keywords:** Completion lobectomy, Video-assisted thoracoscopic surgery, Segmentectomy, Local recurrence, Second primary lung cancer

## Abstract

**Background:**

Completion lobectomy long after segmentectomy in the same lobe is extremely difficult because of severe adhesions around hilar structures, especially in cases involving video-assisted thoracoscopic surgery (VATS) completion lobectomy. We report and compare the surgical outcomes of patients who underwent VATS or thoracotomy completion lobectomy long after radical segmentectomy for lung cancer.

**Methods:**

We retrospectively evaluated the surgical outcomes of completion lobectomies performed at our institute long after radical segmentectomies for lung cancer in the same lobe. The efficacy and safety of VATS completion lobectomy was compared to that of thoracotomy completion lobectomy.

**Results:**

Ten of 228 patients who underwent radical segmentectomy for lung cancer between 2009 and 2018 underwent completion lobectomy at least a month after segmentectomy; five patients underwent VATS completion lobectomy. None of the patients underwent VATS left upper completion lobectomy, and conversion to thoracotomy was required in one patient. There were no significant differences between VATS and thoracotomy completion lobectomies in the median operative times (VATS 295 min, thoracotomy 339 min, *p* = 0.55), intraoperative blood loss volumes (VATS 350 mL, thoracotomy 500 mL, *p* = 0.84), intervals between initial segmentectomy and completion lobectomy (VATS 40 months, thoracotomy 48 months, *p* = 0.55), and number of patients with pulmonary artery injury (VATS 1, thoracotomy 2, *p* = 0.49). There was no operation-related mortality.

**Conclusions:**

VATS completion lobectomy long after segmentectomy for lung cancer could be performed without fatal complications unless severe adhesions are observed around each main pulmonary artery.

## Background

Completion lobectomy (CL) involves resection of the remaining pulmonary lobe after wedge resection or segmentectomy. Successful CLs after diagnostic wedge resection or radical segmentectomy have been reported [1–4]. However, CL long after segmentectomy in the same lobe could be complicated by severe adhesions around hilar structures, especially the pulmonary artery.

The number of radical and anatomical pulmonary segmentectomies performed in cases of early lung cancers with peripherally located small-sized tumors has recently been increasing in Japan [5, 6]. On the other hand, a historical lung cancer study group trial showed a tripling in local recurrence rates in cases involving limited resection compared with those involving lobectomy for lung cancer [7]. Furthermore, the increased incidence of a second primary lung cancer has resulted in an increased number of repeated thoracic surgeries being performed on the ipsilateral side after lung cancer segmentectomy [8, 9]. Therefore, CL is considered in cases involving local recurrence and/or a second primary lung cancer and/or metastatic lung cancer after lung cancer segmentectomy.

Few cases of CL long after segmentectomy have been reported [10]. We report and compare the surgical outcomes of patients who underwent video-assisted thoracoscopic surgery (VATS) or thoracotomy CL long after radical segmentectomy for lung cancer.

## Methods

### Patients and data collection

The medical records of 228 patients who underwent radical segmentectomy for lung cancer at our institute were examined. Of the 228 patients, 10 patients (4.4%) required subsequent CL at least a month after the segmentectomies. These 10 patients were included in this study. The patients included five men and five women with a median age of 69.5 (range, 61–80) years. These patients were divided into two groups: those who underwent VATS CL, which was performed after 2008 as we gradually became familiar with VATS procedures, and those who underwent thoracotomy CL. At our institute, the relative inclusion criteria for VATS CL are as follows: no exposure of each main pulmonary artery during a previous segmentectomy, no radical lymph node dissection (ND) of #4R during a previous segmentectomy of the right upper CL, and ND of #5 and/or #4 L during a previous segmentectomy of the left upper CL. The patients with aforementioned NDs were excluded from VATS CL. We performed intraoperative pulmonary artery isolation and taping in patients with severe adhesion around each main pulmonary artery to address injury-induced bleeding due to pulmonary artery injury.

Data on the following variables were collected and evaluated: operative time, intraoperative blood loss volume, interval between initial segmentectomy and CL, pulmonary lobe targeted for CL, sites of previous NDs, reason for CL, extent and degree of adhesions around hilar structures, intraoperative securing of the pulmonary artery, injury to the pulmonary artery, perioperative complications, and mortality. The degree of adhesion was classified as none, mild, or severe. All patients who underwent CL had undergone previous radical segmentectomy for lung cancer via the VATS approach. Based on the previous segmentectomies, seven patients were diagnosed with primary lung cancer and three with metastatic lung cancer with primary malignancies being rectal cancer, bladder cancer, and leiomyosarcoma, respectively (Table 1).

**Table 1:** Characteristics of patients who underwent completion lobectomy at least a month after segmentectomy

	VATS CL (*N* = 5)	Thoracotomy CL (*N* = 5)
Sex (M/F)	3/2	2/3
Age (years) [range]	72 [66–74]	65 [61–80]
Site of previous segmentectomy (left upper/lower; right upper/lower)	0/1; 2/2	3/1; 1/0
Degree of hilum adhesion at previous segmentectomy (none/mild/severe)	3/2/0	4/1/0
Previous superior mediastinal ND	2 (40%)	3 (60%)
Previous diagnosis (PLC/MLC)	3/2	4/1
Previous R margin status (R0/R1)	5/0	4/1
Clinical diagnosis before CL (recurrence/second PLC/metastasis)	3/1/1	1/3/1
Pathological diagnosis after CL (malignancy/benign lesion)	5/0	3/2

Values are expressed as n (%) and median [range]

*CL* completion lobectomy, *ND* node dissection, *PLC* primary lung cancer, *MLC* metastatic lung cancer

The Mann-Whitney U test and chi-square test were used for the comparison of continuous and categorial variables, respectively. The significance level was set at *p* = 0.05. SPSS version 22.0 (SPSS, Inc., Chicago, IL, USA) statistical software was used for statistical evaluations.

### Surgical procedures

The patients who underwent VATS lung resection at our institute were placed in a lateral position on the operating table under general anesthesia with selective lung ventilation. Two thoracoport trocars (15 mm) were placed in the sixth intercostal space (ICS) at the anterior axillary line and in the seventh ICS at the posterior axillary line. Anterolateral mini-thoracotomy (< 60 mm) was performed in the fourth ICS. Three patients underwent VATS CL via the same operative wound as the previous segmentectomy, whereas two patients underwent CL via an operative wound that was different from the one used for the segmentectomy. Thoracotomy CLs were performed via the previous wound in the fourth ICS extended to a 70–130-mm incision. At our institute, initial CLs were performed with a thoracotomy approach, and the indication of VATS CL had been expanded from the lower pulmonary lobe CL to the upper pulmonary lobe CL, because it is more difficult to secure the main pulmonary artery during CL for the upper pulmonary lobe compared to the lower pulmonary lobe.

## Results

Data on surgical outcomes of each patient who underwent VATS or thoracotomy CL at least a month after radical segmentectomy for lung cancer are shown in Table 2 and Table 3. CL was performed in four cases with suspected local recurrence, in four cases with suspicion of a metachronous second primary lung cancer, and in two cases of metachronous metastasis from non-lung primary malignancy in the same lobe that was resected in the previous segmentectomy, without preoperative pathological diagnosis (Table 1). Two patients underwent right upper CL; one patient, right lower CL; one patient, right middle and lower completion bilobectomy due to a pulmonary hilar tumor; and one patient, left lower CL via the VATS approach. In contrast, three patients underwent left upper CL via the thoracotomy approach, including in one patient where VATS was converted to thoracotomy. One patient underwent left lower CL and one patient underwent right upper CL via the thoracotomy approach (Tables 2 and 3). After the CL, malignancy was diagnosed in eight cases. Intraoperative frozen-section diagnosis initially indicated that the lesions of the two remaining cases were also malignant; however, the final pathological diagnoses were benign lesions (Table 1). Patient number 4 who underwent VATS CL and patient number 3 who underwent thoracotomy CL were diagnosed with local recurrence after CL (Tables 2 and 3).

**Table 2:** Data of patients who underwent VATS completion lobectomy long after segmentectomy

No. (Age/sex)	Lobe for CL	Operative time (min)	Intraoperative blood loss volume (mL)	Degree of hilum adhesion	PA taping	Perioperative complications	Previous segmentectomy procedure	Interval between segmentectomy and CL (months)	Chest tube duration (days)	Length of postoperative stay (days)
1 (66/M)	LLL	295	500	Mild	N	None	Lt. S6 seg	18	4	9
2 (70/F)	RLL	389	200	Severe	Y	PA injury	Rt. S7 + 10 seg +ND2a-1	52	3	16
3 (72/M)	RUL	280	300	Severe	N	None	Rt. S2 seg +ND2a-1	45	7	12
4 (74/F)	RLL +RML	279	950	Mild	N	None	Rt. S8 + 9 seg	4	2	12
5 (73/M)	RUL	342	350	Severe	N	Postoperative Af	Rt. S3 seg +ND2a-1	40	2	14

*CL* completion lobectomy, *LLL* left lower lobe, *RUL* right upper lobe, *RML* right middle lobe, *RLL* right lower lobe, *PA* pulmonary artery, *Af* atrial fibrillation, *S6* segment 6, *Lt* left, *Rt* right, *seg* segmentectomy, *ND* node dissection

**Table 3:** Data of patients who underwent thoracotomy completion lobectomy long after segmentectomy

No. (Age/sex)	Lobe for CL	Operative time (min)	Intraoperative blood loss volume (mL)	VATS converted to thoracotomy (reason)	Degree of hilum adhesion	PA taping	Perioperative complications	Previous segmentectomy procedure	Interval between segmentectomy and CL (months)	Chest tube duration (days)	Length of postoperative stay (days)
1 (61/F)	LUL	458	6870	Yes (PA injury and severe hilar adhesion)	Severe	N	PA injury	LUD seg +ND2a-1	108	7	14
2 (69/F)	RUL	201	200	No	Severe	N	Azygos vein injury	Rt. S2 seg +ND1b	77	2	8
3 (64/M)	LUL	339	1020	No	Severe	Y	None	LUD seg +ND2a-1	2	6	13
4 (80/F)	LUL	391	500	No	Severe	N	PA injury	Lt. S1 + 2 seg +ND2a-1	48	1	18
5 (65/M)	LLL	301	160	No	Mild	N	None	Lt. S9 + 10 seg	21	2	8

*CL* completion lobectomy, *LUL* left upper lobe, *LLL* left lower lobe, *RUL* right upper lobe, *PA* pulmonary artery, *LUD* left upper division, *S2* segment 2, *Lt* left, *Rt* right, *seg* segmentectomy, *ND* node dissection

Two patients who previously underwent superior mediastinal ND with lung cancer segmentectomy underwent VATS CL, and three patients who underwent previous superior mediastinal ND with lung cancer segmentectomy underwent thoracotomy CL (Table 1). There were no significant differences between the VATS CL and thoracotomy CL groups with respect to the median operative times (VATS 295 min, thoracotomy 339 min, *p* = 0.55), intraoperative blood loss volumes (VATS 350 mL, thoracotomy 500 mL, *p* = 0.84), intervals between initial segmentectomy and CL (VATS 40 months, thoracotomy 48 months, p = 0.55), the number of patients with severe adhesion around hilar structures (VATS 3, thoracotomy 4, *p* = 0.49), the number of patients who underwent intraoperative securing of the pulmonary artery (VATS 1, thoracotomy 1), the number of patients with an injury to the pulmonary artery (VATS 1, thoracotomy 2, *p* = 0.49), and chest tube duration (VATS 3 days, thoracotomy 2 days, *p* = 0.69). The patient with pulmonary artery injury during VATS CL underwent pulmonary artery occlusion with a 1–0 silk suture, and suturing of the pulmonary artery was performed via the VATS approach without converting to thoracotomy. There was no operation-related mortality (Table 4). In the case of Patient 3, who underwent thoracotomy CL, we opened the pericardium and performed intrapericardial pulmonary artery taping to prevent catastrophic bleeding caused by severe adhesion around the main pulmonary artery and left upper bronchus.

**Table 4:** Summarized surgical outcomes of patients who underwent completion lobectomy long after segmentectomy

	VATS CL (*N* = 5)	Thoracotomy CL (*N* = 5)	*p*-value
Pulmonary lobe targeted for CL	LUL 0	LUL 3 (60%)	
	LLL 1 (20%)	LLL 1 (20%)	
	RUL 2 (40%)	RUL 1 (20%)	
	RLL 1 (20%)	RLL 0	
	RML + RLL 1 (20%)	RML + RLL 0	
Operative time (min) [range]	295 [279–389]	339 [201–458]	0.55
Intraoperative blood loss volume (mL) [range]	350 [200–950]	500 [160–6870]	0.84
Severe hilum adhesion	3 (60%)	4 (80%)	0.49
PA taping	1 (20%)	1 (20%)	
PA injury	1 (20%)	2 (40%)	0.49
Chest tube duration (days) [range]	3 [2–7]	2 [1–7]	0.69
Interval between segmentectomy and CL (months) [range]	40 [4–52]	48 [2–108]	0.55
Operation-related mortality	0	0	

Values are expressed as n (%) and median [range]

*CL* completion lobectomy, *LUL* left upper lobe, *LLL* left lower lobe, *RUL* right upper lobe, *RML* right middle lobe, *RLL* right lower lobe, *PA* pulmonary artery

## Discussion

The present study shows that the surgical outcomes of VATS CL and thoracotomy CL were not significantly different. Generally, the VATS approach is superior to thoracotomy in terms of magnifying the operative field, knowledge of the surgical technique, reduction of postoperative pain, and faster recovery of postoperative pulmonary function [11]. Nevertheless, VATS CL long after segmentectomy in the same lobe has not been reported because it is extremely difficult due to severe hilar adhesions. In Japan, radical and anatomical pulmonary segmentectomies are actively performed in cases involving early lung cancers with peripherally located small-sized tumors. A prospective study reported no recurrence of primary lung cancer during a 5-year follow-up period after limited resection of lung cancer with a maximum tumor diameter of 8–20 mm, a ground glass opacity (GGO) ratio > 80% on computed tomography, and clinical T1N0M0 classification. Moreover, the 5-year disease-specific and overall survival rates were 100 and 98%, respectively [6].

At our institute, radical and anatomical pulmonary segmentectomies are performed in cases involving lung cancer with peripheral pulmonary nodules < 20 mm in diameter and a GGO ratio > 50% on computed tomography. Preservation of lung function after segmentectomy results in a large amount of resectable metachronous second primary lung cancer in the non-resected lobe after segmentectomy of the initial primary lung cancer [8, 9]. On the other hand, the detection of new solitary pulmonary nodules during postsurgical follow-up in the previously resected lobe, especially after limited resection, poses a diagnostic challenge. At our institute, intraoperative frozen-section diagnosis is performed to decide which surgical treatment is needed for lesions, without preoperative pathological diagnosis, suspected to be recurrent and/or second primary lung cancer after lung cancer segmentectomy. Nevertheless, CL is necessary when intraoperative frozen section diagnosis indicates malignancy during surgeries for lesions suspected of local recurrence and/or second primary lung cancer. CL is one of the treatments for patients with local recurrence and/or a second primary lung cancer and/or metastasis after lung cancer segmentectomy. In our study, CL was performed in four cases with suspected local recurrence, in four cases with a suspicion of a metachronous second primary lung cancer, and in two cases with metachronous metastasis from non-lung primary malignancy in the same lobe as the one previously resected (Table 1).

CL long after segmentectomy is difficult because mobilization of the hilum structure is challenging owing to dense adhesions around it that have already been divided and manipulated during the previous segmentectomy. In fact, it was reported that CL may become more difficult to perform approximately 5 weeks after segmentectomy [10]. In our study, CLs were performed at least a month after the previous lung cancer segmentectomies, and hilum adhesion was especially severe after superior mediastinal ND during the previous lung cancer segmentectomies (Tables 2 and 3).

Taping and/or clamping of the main pulmonary artery is occasionally needed to prevent catastrophic bleeding when it is difficult to expose and divide the pulmonary artery because of hilum adhesion. At our institute, we expose the pulmonary artery enough to clamp with forceps for the cases with severe hilum adhesion, and taping is performed in the case that pulmonary artery is completely isolated. It is more difficult to secure the pulmonary artery and/or arrest bleeding during the VATS approach than during the thoracotomy approach when bleeding occurs from the pulmonary artery. We think that VATS CL is safe to perform if each central main pulmonary artery is secured by the VATS approach. At our institute, pulmonary artery occlusion using silk suture (double looping technique: DLT) has been performed for securing them during the VATS approach [12]. From this point of view, we consider that our exclusion criteria for VATS CL, which may cause difficulty in using DLT, are appropriate. Opening of the pericardium and intrapericardial main pulmonary artery taping, which are needed to address severe hilum adhesion around the superior vena cava and main pulmonary artery in right CL, are difficult to perform safely with the VATS approach. On the other hand, it is also difficult to isolate the main pulmonary artery in patients with adhesion around the ligamentum Botalli, which often needed to be cut before the main pulmonary artery can be isolated. Nevertheless, the length of the left central pulmonary artery is longer than the right. In this study, two of the 10 patients who underwent CL received pulmonary artery taping. One patient underwent main pulmonary artery taping with a silk suture during VATS CL, and the other underwent intrapericardial pulmonary artery taping during the thoracotomy CL (Tables 2 and 3). The patient with pulmonary artery injury underwent pulmonary artery occlusion with a silk suture, and the suturing was performed via the VATS approach, without conversion to thoracotomy.

We suggest that VATS CL long after segmentectomy for lung cancer can be performed without fatal complications; however, we experienced a case wherein VATS was converted to thoracotomy for a left upper CL to arrest bleeding from the main pulmonary artery (Table 3). In this patient, exposing and securing the main pulmonary artery was technically difficult during the VATS approach because of severe adhesion around the main pulmonary artery and aorta, though it was possible to arrest the bleeding by using the VATS approach.

There are several limitations associated with this study. First, the study included very few patients; therefore, the lobes that underwent previous segmentectomy and the regions in which ND was carried out were inconsistent during VATS and thoracotomy CL. However, we conclude that because of severe hilum adhesions, CL may become more difficult after radical superior mediastinal ND. Second, the present study had a retrospective design. Although it may be difficult to conduct a large-scale and prospective study of CL, many reports of CL will be necessary to acquire robust evidence to support the safety of VATS CL.

## Conclusions

In conclusion, VATS CL in the same lobe long after radical segmentectomy for lung cancer could be performed without fatal complications in selected patients. However, VATS CL should be avoided in patients with previous radical superior mediastinal ND; such cases complicate exposing and clamping the main pulmonary artery with the VATS approach as well as use of DLT and may require an intrapericardial pulmonary artery clamp.

## Data Availability

The datasets used and/or analyzed during the current study are available from the corresponding author on reasonable request.

## Abbreviations

CL: Completion lobectomy
DLT: Double looping technique
GGO: Ground glass opacity
ICS: Intercostal space
ND: Node dissection
VATS: Video-assisted thoracoscopic surgery
